# Cystamine reduces neurodegeneration and epileptogenesis following soman-induced status epilepticus in rats

**DOI:** 10.3389/ebm.2025.10598

**Published:** 2025-06-09

**Authors:** Abiel K. Biney, Caroline R. Schultz, Michael F. Stone, Donna A. Nguyen, Annie Wang, Marcio de Araujo Furtado, Lucille A. Lumley

**Affiliations:** ^1^ Neuroscience Department, U.S. Army Medical Research Institute of Chemical Defense (USAMRICD), Aberdeen, MD, United States; ^2^ BioSEaD, LLC, Rockville, MD, United States

**Keywords:** organophosphorus nerve agents, seizures, status epilepticus, cystamine, neuroprotection

## Abstract

Acute exposure to a seizure-inducing dose of an organophosphorus nerve agent inhibits acetylcholinesterase, leading to pharmacoresistance if benzodiazepine treatment is delayed. Following soman-induced status epilepticus (SE) in rats, prolonged seizure is associated with severe and widespread neurodegeneration. We evaluated the aminothiol cystamine, the oxidized form of cysteamine, for neuroprotective potential against soman-induced SE and associated neurodegeneration. Cystamine has a myriad of effects including antioxidant properties, neuroprotective effects, and immunomodulation, among others, which is of interest in evaluating neuroprotective efficacy against cholinergic-induced neurodegeneration. Adult male rats implanted with telemetry transmitters for continuous EEG recording were exposed to soman and treated with the muscarinic antagonist atropine sulfate and the oxime asoxime dimethanesulfonate 1 min after exposure to increase survival. Midazolam was administered 30 min after seizure onset. Cystamine (10 or 50 mg/kg) or vehicle was administered 30 min after seizure onset and again 4 h after soman exposure. The initial seizure duration, the EEG power integral at 6 h after exposure, and the percentage of rats that developed spontaneous recurrent seizure were reduced in rats treated with cystamine, compared to those that received only midazolam. In addition, cystamine reduced neurodegeneration in seizure-sensitive brain regions following soman exposure, compared to midazolam. Our findings highlight the potential for aminothiols to serve as adjunctive therapy to midazolam in treating cholinergic-induced toxicity and suggest broader applications of aminothiols in neuroprotection and neurological disorders.

## Impact statement

Current research faces challenges in addressing long-term neurodegeneration. Using a preclinical model of cholinergic-induced status epilepticus and associated neurodegeneration, our study investigated the neuroprotective potential of the aminothiol cystamine, demonstrating its ability to reduce chronic neuropathology when used as an adjunct to the current standard of care. The findings presented here not only emphasize the efficacy of cystamine in mitigating cholinergic-induced neurodegeneration, but also suggest its potential role in reducing initial time in seizure, neuroinflammation and epileptogenesis. Our findings contribute valuable insight into the broader applications of aminothiols in neuroprotection.

## Introduction

Recent studies suggests that aminothiols, such as cystamine and cysteamine, may serve as promising novel neuroprotectants (reviewed in Paul and Snyder, 2019) [1]. The aminothiol cystamine is the oxidized form of cysteamine, which is a decarboxylated derivative of cysteine. Cysteamine is FDA-approved for use in treatment of nephropathic and ocular cystinosis; cystinosis is a genetic disorder in which cystine crystals build up in tissues. More recently, cysteamine and cystamine, are under evaluation for potential treatment of neurodegenerative disorders. Cystamine and cysteamine are recognized for their antioxidant properties, capable of scavenging harmful free radicals and mitigating oxidative stress as observed in emerging studies investigating neurodegenerative diseases like Huntington’s, Alzheimer’s, and Parkinson’s disease (reviewed in Paul and Snyder, 2019) [1]. Cystamine’s inhibition of transglutaminase, an enzyme linked to neuronal death, along with its ability to reduce oxidative stress has shown to preserve neuronal integrity in Huntington’s disease models, highlighting its protective role against neurodegeneration [2, 3]. Similarly, its analog cysteamine reduced the toxic effects of mutant huntingtin proteins by modulating mitochondrial dysfunction and preventing apoptosis [4]. Both aminothiols increase brain-derived neurotrophic factor (BDNF) levels in mouse brain, enhancing neuronal survival and repair [2, 5, 6]. Calkins et al. (2010) [7] reported that cystamine attenuated oxidative damage in a neurotoxicity model involving exposure to 3-nitropropionic (3-NP) acid, a neurotoxin that induces mitochondrial dysfunction, similar to that observed in Parkinson’s disease. Cystamine and cysteamine have demonstrated efficacy in rescuing dopaminergic neurons and promoting restorative mechanisms in rodent models of Parkinson’s disease and 1-methyl-4-phenyl-1,2,3,6-tetrahydropyridine (MPTP)-induced toxicity [6, 8, 9], with cysteamine further identified as a potential disease-modifying agent agent that improved cognitive and motor function, and delayed disease progression in mice (reviewed in Cicchetti et al., 2019 [10]). While much of the current research on cystamine treatments centers on neurological conditions that are progressive and long-term in nature, this study investigates the acute effects of cystamine. The findings presented here may suggest its potential application in mitigating neurological injury and disease, but further research to include long-term studies are needed. We evaluated cystamine for its neuroprotective efficacy in a rat model of cholinergic-induced status epilepticus and associated neurodegeneration caused by organophosphorus nerve agent exposure.

Organophosphorus nerve agents are potent, fast acting irreversible inhibitors of acetylcholinesterase, which, if left untreated, rapidly lead to a cholinergic crisis and fatality due to respiratory failure [11, 12]. In preclinical studies, exposure to a seizure-inducing dose of nerve agent, such as soman, resulted in long-term, progressive pathophysiology characterized by neurodegeneration, epileptogenesis, neuroinflammation, and neurological dysfunction [13–17]. This pathological progression is also demonstrated by the onset of status epilepticus, a condition marked by recurrent seizures that impede neurological recovery and exacerbate long-term damage [15, 16, 18]. Chen et al. (2012) [17] and Jett et al. (2020) [19] discussed findings in human and animal studies, where disruptions to the cholinergic system caused by nerve agent exposure is linked to increased levels of oxidative stress and persistent cognitive deficits such as impaired learning, memory loss, mood disturbances, and attention deficits. The current therapeutic standard of treatment for nerve agent exposure includes the muscarinic antagonist atropine, an oxime such as 2-pralidoxime, and a first-line benzodiazepine such as midazolam or diazepam [20, 21]. Although this therapeutic strategy increases survival, delayed treatment, which is anticipated in a mass casualty event, may result in benzodiazepine refractory status epilepticus and failure to fully protect against the neuroinflammation and neurodegeneration induced by status epilepticus [22–24]. These events underscore the need for improved medical and public health preparedness, particularly in mass casualty scenarios.

Considering current drug limitations and lack of approved neuroprotectants to mitigate the secondary neurodegeneration that follows nerve agent exposure, we evaluated the potential neuroprotective efficacy of the aminothiol cystamine as a novel adjunct to midazolam against soman-induced status epilepticus in rats. The organophosphorus nerve agent soman was used to assess the neuroprotective efficacy of cystamine, since in preclinical models, exposure to a lethal dose of soman consistently induces seizure [24, 25] and seizure-associated brain damage [26, 27]. Soman’s rapid induction of seizure activity makes it more reliable for neuroprotection studies compared to nerve agent VX, which although highly toxic, has less consistent SE compared to soman [24, 28]. In addition, the most effective anticonvulsant drugs in soman-induced seizures are either equally or more effective in treating seizures induced by other nerve agents (tabun, sarin, cyclosarin, VX) [24], suggesting therapies effective against soman may be broadly applicable to other nerve agents. Soman is difficult to treat since it undergoes a rapid “aging” process, within minutes [29], whereby nerve agent inhibited acetylcholinesterase (AChE) is converted to an inactive form, limiting the window for oxime reactivation. In addition, in rats exposed to soman, the U.S. fielded oxime pralidoxime chloride (2-PAM) afforded no protection [30].

Early control of nerve agent-induced seizure is critical for both survival and neuroprotection [24] as there is a time-dependent reduction in efficacy of benzodiazepines in terminating nerve-agent induced seizure [31, 32]. Our laboratory developed a preclinical rat model of soman-induced seizure to evaluate therapies to treat SE, epileptogenesis and associated neurodegeneration [18, 33–36]. Soman’s reliability in producing severe, benzodiazepine-resistant SE in rats makes it particularly valuable for studying neuroprotection in refractory SE scenarios. In sum, the well characterized preclinical rat model of soman-induced SE results in benzodiazepine refractory SE and severe neuropathology, making it a reliable and rigorous model for testing therapeutics against cholinergic-induced seizure and associated neurodegeneration. Animal studies conducted in our laboratory using this preclinical model, along with evidence in literature suggests that aminothiols may serve as promising novel adjunctive therapies for counteracting nerve-induced neurodegeneration, neuroinflammation, and epileptogenesis.

## Materials and methods

### Animals

Adult male Sprague Dawley rats (276–300 g; Charles River Laboratories; Kingston, NY, United States) were pair-housed upon arrival and then housed in individual cages at the time of surgery. Animals were kept in temperature and humidity-controlled quarters with food and water available *ad libitum*. Rooms were set on a standard 12:12 h light-dark cycle (lights on at 0600) and rats were weighed daily (M-F) with exception of weekends and federal holidays. The experimental protocol was approved by the Animal Care and Use Committee at the United States Army Medical Research Institute of Chemical Defense, an AAALAC accredited facility, and all procedures were conducted in accordance with the principles stated in the Guide for the Care and Use of Laboratory Animals and the Animal Welfare Act of 1966 (P.L. 89–544), as amended.

### Telemetry transmitter implantation for electroencephalographic recording

Rats were implanted subcutaneously (SC) with electroencephalographic (EEG) telemetry transmitters. For pain management, rats were pretreated with meloxicam (1 mg/kg, SC; Patterson Veterinary; St. Paul, MN, United States) prior to being anesthetized with isoflurane (Patterson Veterinary; 5% chamber induction, 1–5% maintenance with 0.5–10 L/oxygen). Once rats were secured in a Kopf stereotaxic apparatus (David Kopf Instruments, Tujunga, CA, United States), four stainless steel screw electrodes were implanted cortically through the skull 2 mm from each side of the midline at 2 mm anterior and 4 mm posterior to bregma. HD-S02 or F40-EET transmitters (Data Sciences International [DSI], Inc., St. Paul, MN, United States) were implanted SC, with the ends of the four stainless-steel wires wrapped around each screw electrode. Electrodes and wrapped wires were secured in place with self-curing dental acrylic (Ortho-Jet™, Lang Dental Manufacturing Company, Inc., Wheeling, IL, United States). Buprenorphine sustained release (SR) or extended release (ER) (1.2 mg/kg, SC; ZooPharm, Laramie, WY, United States) was administered as a post-operative analgesic immediately after removal from anesthesia. Rats were given food pellets dissolved in water with a few grains of sugar to aid with post-operative recovery and at least 1 week of surgical recovery prior to experimentation. EEG activity was continuously recorded using the Ponemah V6 or Dataquest Art Acquisition (digitized at 500 Hz, DSI, Inc.).

### Agent exposure and treatments

Rats were exposed to saline (Hospira; Lake Forest, IL, United States) or a seizure-inducing dose of soman (GD; 0.5 mL/kg, 236.2 μg/mL, SC; obtained from the United States Army Combat Capabilities Development Command Chemical Biological Center; Aberdeen Proving Ground, Gunpowder, MD, United States) then treated with an admix of atropine sulfate (ATS; 2 mg/kg, IM; Sigma-Aldrich; St. Louis, MO, United States) and asoxime dimethanesulfonate (HI-6 in DMS; 118.5 mg/kg, IM, Kalexyn Medicinal Chemistry, Kalamazoo, MI, United States) 1 min after exposure, followed by midazolam (3 mg/kg, SC; Hospira) 30 min after seizure onset. In our study, we administered a dose of 1.2 LD_50_ soman which is approximately the LD_90_ for subcutaneous exposure in untreated adult male rats (data generated in our laboratory) [37]. The ATS and HI-6 admix treatments were administered to simulate the standard medical response following exposure and to increase survival. Controls (No GD) did not receive the admix. EEG recordings were monitored in real time to identify the onset of seizure defined as rhythmic, high amplitude spikes (>2 × baseline values) lasting ≥10 s.

Cystamine (10 or 50 mg/kg, IP) or vehicle was administered 30 min after seizure onset and again 4 h later. The experimental setup consisted of four groups: control (No GD, n = 14), midazolam (GD/MDZ/VEH, n = 14), midazolam-cystamine 10 mg/kg (GD/MDZ/CYS10, n = 7), and midazolam-cystamine 50 mg/kg (GD/MDZ/CYS50, n = 10). Cystamine given at doses of 10 and 50 mg/kg were previously shown to be neuroprotective in rodents (10, 50, and 100 mg/kg [38]; 30 and 60 mg/kg [39]). Both studies administered cystamine repeatedly over multiple weeks. For the present study, we gave a single additional dose of cystamine. Seven out of the fourteen rats in the control group and eight out of the fourteen rats in the midazolam group are historical controls. Three rats from the control (No GD) group had nonfunctional transmitters (no remaining battery life) and were excluded from temperature, activity, and power band analysis. Histological analysis for NeuN was performed for a subset of n = 11 rats in the control (No GD) group. Histological analysis for Iba1 was performed for a subset of n = 11 rats in the control (No GD) group. Data missing values because of death, include one rat in the cystamine (10 mg/kg) group that died after nerve agent exposure before receiving the second treatment and was excluded from all data analysis. One rat in the cystamine (10 mg/kg) group was euthanized early for humane endpoint 4 days after exposure and excluded from body weight, spontaneous recurrent seizures (SRS), activity, and neuropathological analysis. One rat in the cystamine (50 mg/kg) group did not survive to 24 h and was excluded from all analysis except toxic signs and power integral.

### Analysis of EEG seizure and behavioral seizure

Continuous recording of EEG, temperature, and activity data were recorded using the Ponemah software (DSI, Inc.) and Dataquest Art Acquisition (DSI, Inc.) for subjects with HD-S02 and F40-EET transmitters. Home cages were placed on RPC-1 PhysioTel receivers (DSI, Inc.), where baseline EEG was recorded at least 1 day prior to exposure and continuously for 14 days after exposure. The EEG power spectrum was divided into delta (0.1–4.0 Hz), theta (4.1–8.0 Hz), alpha (8.1–12 Hz), beta (12.1–25 Hz), and gamma (25.1–50 Hz) bands. The mean power was calculated for each band (see de Araujo Furtado et al., 2009 for methods) [40] and integrated in 10-min bins. Calculation of the EEG power integral to measure seizure severity was determined by taking the average of power spectra of each hour period using a customized MATLAB algorithm and applying a formula {decibels = 10*[Log(V^2^sample/V^2^baseline)]}*60 min, resulting in decibels/h (see Niquet et al., 2016 and Lumley et al., 2019 for methods) [41, 42]. The frequency range was 0.1–100 Hz, with the data representing the full spectrum and the ratio of EEG power in specific time periods after onset of SE or treatment. Noldus Observer XT (Noldus Information Technology Inc., Leesburg, VA, United States) was used for real-time monitoring and to input behavioral seizure continuously for 2 h after exposure and then every 30 min until close of business. A modified Racine scale [43] was used to score behavioral seizures, comprised of the following five stages: 1, masticatory movements; 2, head myoclonus; 3, limb clonus and/or tonus; 4, forelimb clonus with rearing; 5, rearing and falling and/or tonic-clonic convulsions. EEG was also monitored throughout this observation period. Additional sterile saline (5 mL, SC) treatments and food pellets dissolved in water with grains of sugar were provided 1-2 times daily to aid with weight recovery and dehydration for the first 3-4 days after exposure.

### Neuropathology assessments

Two weeks after soman exposure, rats were deeply anesthetized with sodium pentobarbital (75 mg/kg, 1.0 mL, IP; Euthasol, Virbac or Fatal-Plus, Patterson Veterinary) and then transcardially exsanguinated with 0.9% heparinized saline in 0.1 M phosphate buffer (PB; FD Neurotechnologies, Columbia, MD, United States), followed by 4% paraformaldehyde in 0.1 M PB (FD Neurotechnologies) for tissue fixation. Following perfusions, brains were removed and post-fixed in 4% paraformaldehyde for 6 h at 4–8°C then cryoprotected in 20% sucrose (FD Neurotechnologies) in PB for up to 1 week. Brains were later flash frozen at −70°C in preparation for sectioning and staining. Brain tissue was sectioned coronally at 30 µm and stained with mature neuronal marker NeuN (mouse anti-NeuN IgG 1:1000; Millipore, Billerica, MA, United States) or microglial marker ionized calcium-binding adaptor molecule 1 (Iba1; rabbit antiIba1 IgG 1:10,000; Wako Chemicals, Richmond, VA, United States). Processing for the stains was conducted by FD Neurotechnologies. Viable neurons were quantified through analysis of NeuN-positive (NeuN+) cells and neuroinflammation was quantified through analysis of Iba1-positive (Iba1+) cell density and cell body-to-size ratio in the following brain regions, which our laboratory and others [21, 33, 36] previously observed soman-induced neurodegeneration: layer 3 of the piriform cortex, CA1 of the hippocampus, amygdala, medial thalamus, and lateral thalamus.

An Olympus brightfield microscope and VSI slide scanning software were utilized to obtain images of slides. NeuN-stained coronal sections (30 μm, scanned at ×10 magnification, slides between Bregma −2.40 to −3.24 mm) were analyzed using ImagePro v7.0 (Media Cybernetics, Inc., Rockville, MD, United States) to evaluate NeuN-positive (NeuN+) cell density and density values (areas measured in µm) were obtained using automated counts. Inverted contrast was applied to aid in the visualization and counting of NeuN+ cells. StereoInvestigator (MBF Biosciences, Williston, VT, United States) was utilized to manually count NeuN+ cell densities in the highly dense region of the CA1 of the hippocampus. Iba1-stained coronal sections (30 μm, scanned at ×20 magnification, slides between Bregma −2.40 to −3.24 mm) were analyzed in ImageJ (National Institutes of Health, NIH, Bethesda, MD, United States) for Iba1-positive (Iba1+) cell density and Iba1+ cell body-to-size ratio to evaluate microglial activation. Control (No GD) subjects served as comparisons for neuropathology evaluation; subjects that did not survive until study endpoint of 14 days after exposure were excluded from neuropathological analysis.

### Data analysis

Statistical analyses were performed using SPSS (IBM Inc. Armonk, NY, United States) and graphs generated using Prism GraphPad 10 (Dotmatics; Boston, MA, United States). For group comparisons of body weight, temperature, activity, EEG power band, and EEG power integral, repeated measures analysis was used followed by an analysis of variance with a Tukey’s test. For comparisons of initial seizure duration, NeuN+ density, and Iba+ density and ratio, a one-way ANOVA was done followed by Tukey’s test. Treatment effect on median latency to SRS onset was determined with a Kaplan-Meier analysis. The effect of treatment on the endpoint percent of subjects that developed SRS was determined using a binary logistic regression analysis followed by a Chi-Square Fisher’s exact test. The effect of group on the number of SRS occurrences was determined based on a negative binomial model. Behavioral seizure score was analyzed using a Kruskal-Wallis test.

## Results

### Body weight, body temperature, and home cage activity after soman exposure

**FIGURE 1:**
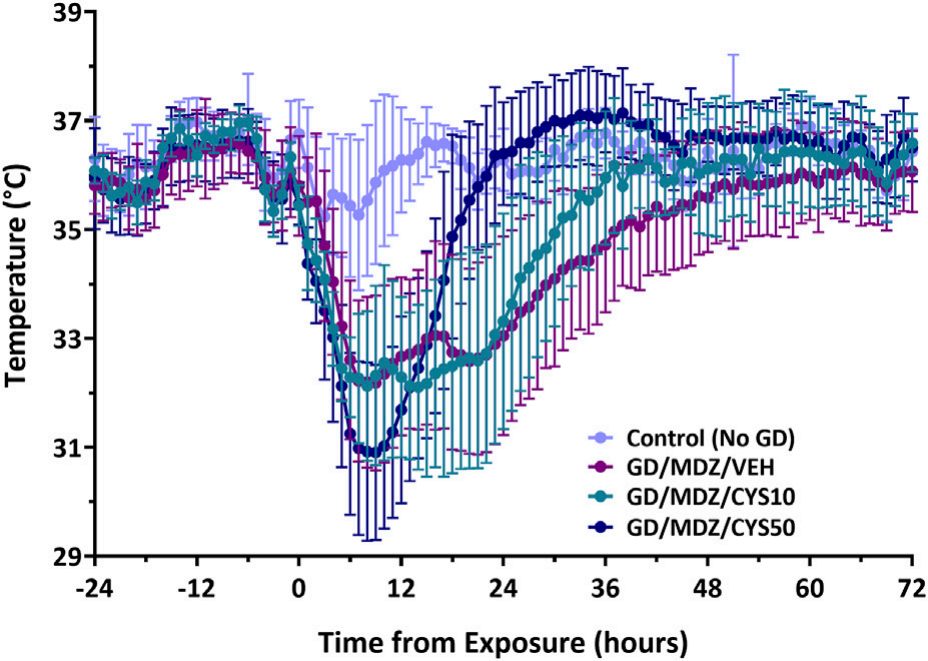
The effect of cystamine treatment on body temperature in adult male rats exposed to soman. Rats exposed to soman were treated with atropine sulfate and HI-6 1 minute after exposure and with midazolam (GD/MDZ/VEH) with or without 10 mg/kg cystamine (GD/MDZ/CYS10) or 50 mg/kg cystamine (GD/MDZ/CYS50) 30 min and 4 h after seizure onset. In all soman-exposed groups, body temperature was reduced within a few hours compared to baseline and to Control (No GD). The GD/MDZ/CYS50 group had lower body temperature at 1 h and 2 h compared to the GD/MDZ/VEH group (p < 0.05), yet recovered body temperature more rapidly; from 18 to 44 h, 46–48 h, and 56 h after exposure. The GD/MDZ/CYS50 rats had significantly higher body temperature compared to GD/MDZ/VEH (p < 0.05). In addition, the GD/MDZ/CYS50 group returned to baseline temperature by 18 h, the GD/MDZ/CYS10 within 26 h and the GD/MDZ/VEH within 37 h after exposure. n = 11 for Control (No GD), n = 14 for GD/MDZ/VEH, n = 6 for GD/MDZ/CYS10, n = 9 for GD/MDZ/CYS50. Data are shown as mean ± SD.

Five out of the seven rats treated with the 10 mg/kg dose and nine out of the ten rats treated with the 50 mg/kg of cystamine survived exposure to soman. All rats that received midazolam monotherapy survived exposure to soman and only surviving historical rats were included. All soman-exposed groups experienced a reduction in body weight within 24 h of exposure (p < 0.05; Supplementary Figure S1). Body weight in soman-exposed rats that received cystamine (50 mg/kg) as adjunct to midazolam did not differ from control rats in body weight by post-exposure day 3, whereas those that received midazolam monotherapy weighed less than control rats until 1 week after exposure. Following soman exposure, rats experienced a decline in body temperature in the first few hours after exposure, with temperatures stabilizing close to baseline levels after approximately 36–42 h after exposure (Figure 1). While all three soman-exposed groups experienced a drop in body temperature following exposure, the temperatures of the group that received 50 mg/kg of cystamine returned to baseline within 18 h of exposure while those that received 10 mg/kg of cystamine returned within 26 h, and those that received midazolam monotherapy returned within 37 h of exposure (p < 0.05). In addition, rats treated with cystamine (50 mg/kg) and midazolam had higher temperature compared to midazolam monotherapy, from 18–44, 46–48 and at 56 h after soman exposure. The group that received 10 mg/kg of cystamine returned to baseline within 29 h.

Home cage activity increased in the dark cycle of soman-exposed rats treated with either midazolam or with the lower dose of cystamine (10 mg/kg), compared to control (No GD) rats (p < 0.05; Supplementary Figure S2). Significance was found in rats treated with cystamine (10 mg/kg) during the dark cycle on days 8 and 9 and in rats treated with midazolam-only on days 10 and 11 following exposure compared to controls. Rats that received midazolam and cystamine (10 mg/kg) also had increased activity on days 2-3 after exposure. In contrast, activity in rats treated with the higher dose of cystamine (50 mg/kg) as adjunct to midazolam did not differ from control.

### Behavioral seizure, EEG seizure activity, power integral, and epileptogenesis

**FIGURE 2:**
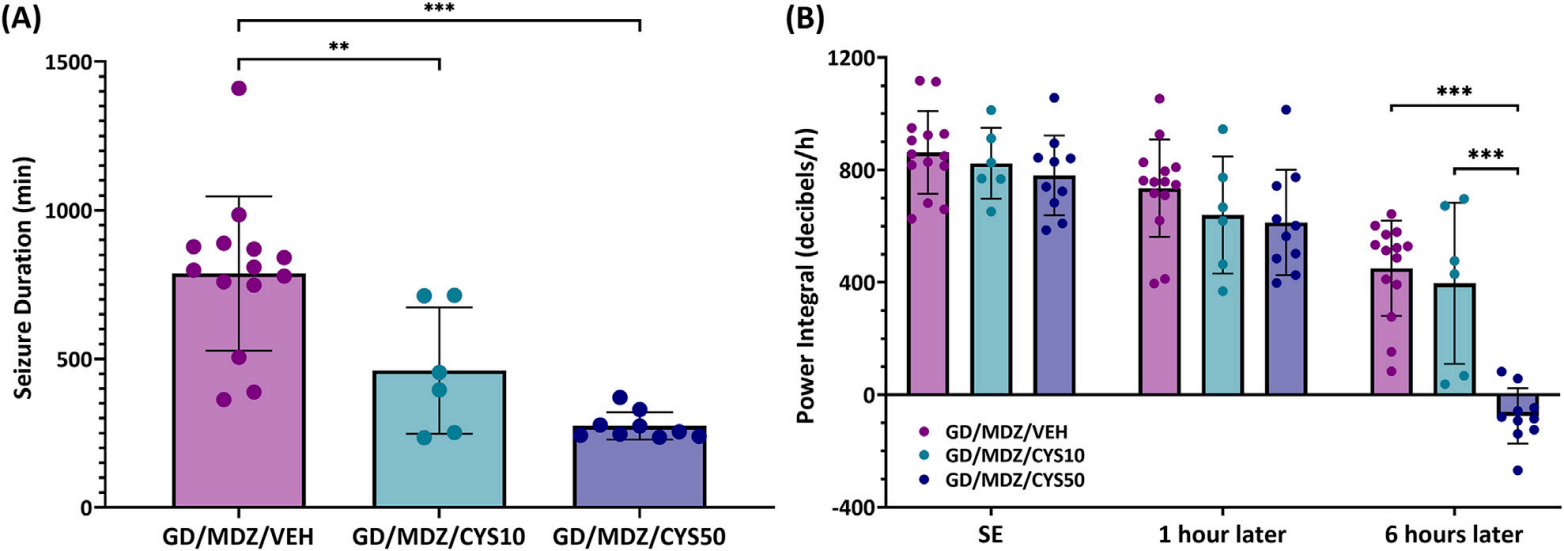
The effect of cystamine treatment on initial seizure duration and power integral in adult male rats exposed to soman. Rats exposed to soman were treated with atropine sulfate and HI-6 1 minute after exposure and with midazolam (GD/MDZ/VEH) with or without 10 mg/kg cystamine (GD/MDZ/CYS10) or 50 mg/kg cystamine (GD/MDZ/CYS50) 30 min and 4 h after seizure onset. **(A)** Adult male rats exposed to soman and treated with the cystamine (10 or 50 mg/kg) had reduced duration of initial seizures during the first 24 h compared to midazolam-treated rats (***p < 0.001; **p < 0.01). n = 14 for GD/MDZ/VEH, n = 6 for GD/MDZ/CYS10, n = 9 for GD/MDZ/CYS50. **(B)** Comparison of power integral at SE onset, 1 h after treatment, and 6 h after treatment. Power integral is averaged over 10 min time bins. Of the cystamine groups, neither showed differences from midazolam monotherapy at 1 h. Only the 50 mg/kg dose showed a significant reduction in the EEG power integral at the 6 h post-treatment period compared to midazolam monotherapy and compared to the lower cystamine dose (***p < 0.001). n = 14 for GD/MDZ/VEH, n = 6 for GD/MDZ/CYS10, n = 10 for GD/MDZ/CYS50. Data are shown as mean ± SD.

All soman-exposed adult rats experienced moderate-to-severe behavioral seizure within minutes of exposure. Animals exhibited behavioral seizure corresponding to stages 4-5 within the first hour following exposure and signs persisted at stages 2-3 over the 6-h period after exposure. Neither dose of cystamine dose as adjunct with midazolam reduced severity of behavioral seizures shortly after treatment compared to midazolam monotherapy; however, the 50 mg/kg dose of cystamine had significantly reduced behavioral seizure scores at the 210-min time point (p < 0.05). Acute (24 h) seizure duration and EEG power integral were monitored in adult rats that had received telemetry implants 7–10 days prior to soman exposure. Among rats treated with the cystamine, the duration of initial seizures during the first 24 h was significantly reduced compared to midazolam-only treated rats, with the 50 mg/kg dose showing the greatest reduction (p < 0.001; Figure 2A). In addition, the 50 mg/kg dose of cystamine reduced EEG power integral 6 h post-treatment when compared to midazolam alone (p < 0.001; Figure 2B).

**FIGURE 3:**
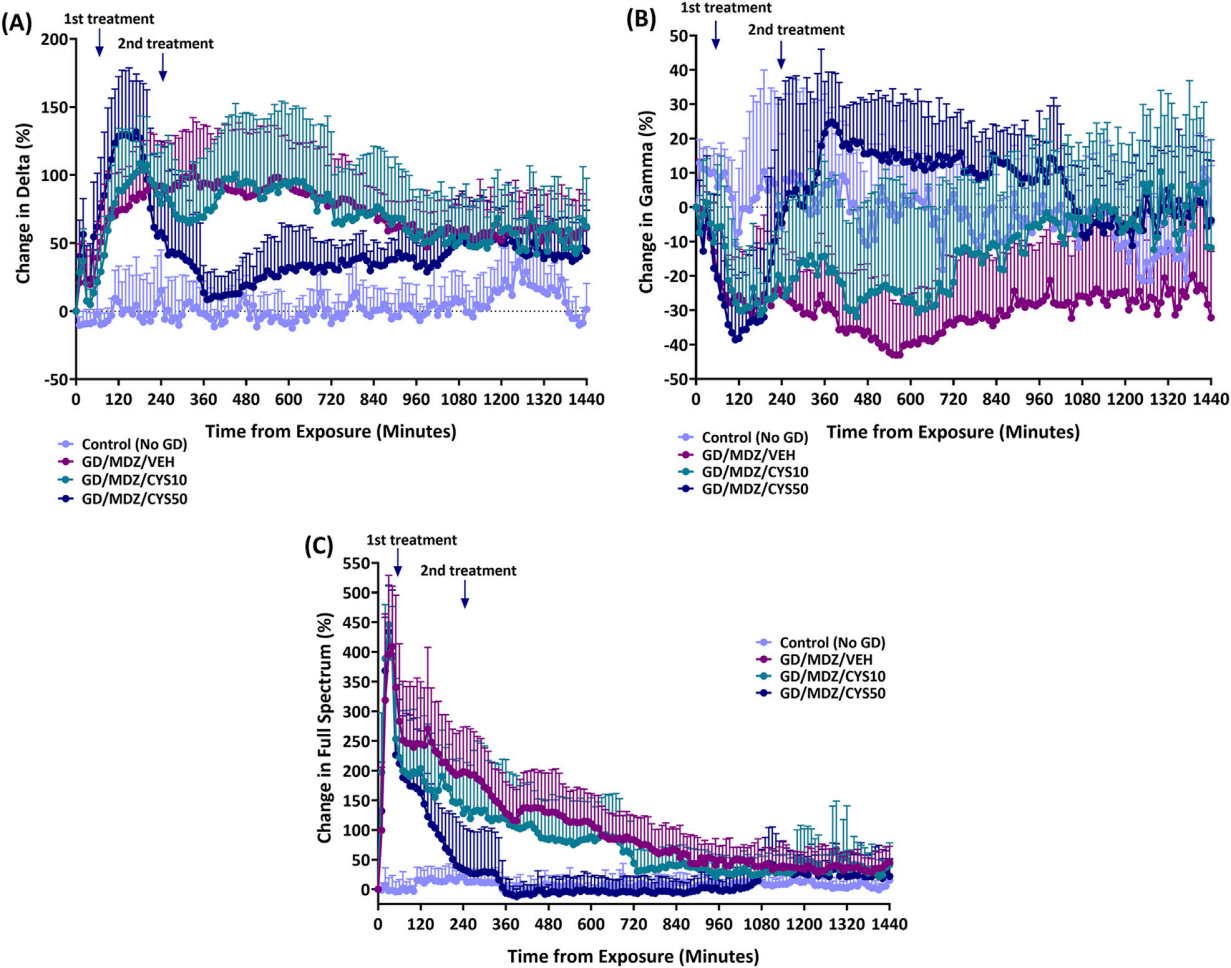
The percent of relative change from baseline levels in the power of **(A)** delta band (0.1–4 Hz), **(B)** gamma band (25.1–50 Hz), and **(C)** total power spectra was calculated for soman-exposed rats that received midazolam (GD/MDZ/VEH) or cystamine as adjunct to midazolam (GD/MDZ/CYS10; GD/MDZ/CYS50). The GD/MDZ/CYS50 group had reduced change in delta band activity, increased change in gamma band, and reduced change in total EEG power spectra compared to soman-exposed rats that received midazolam (GD/MDZ/VEH). **(A)** For delta p < 0.05 in comparison of Control (No GD) vs. GD/MDZ/CYS50 at 10–220, 250, 290, 600–610, 650, 720–770, 800–1000, 1020–1200, 1230, and 1380–1440 min after exposure; GD/MDZ/VEH vs. GD/MDZ/CYS50 at 20, 50, 110–170, 280–880, 960–970, and 990–1000 min after exposure. **(B)** For gamma p < 0.05 in comparison of Control (No GD) vs. GD/MDZ/CYS50 at 10–20, 40–190, 400, 460–480, 500, 680–690, 790, and 850–870 min after exposure; GD/MDZ/VEH vs. GD/MDZ/CYS50 at 260–1070, 1140–1150, 1210, 1230, 1260–1270, 1300, 1320, 1340–1350, 1370, 1400, 1420, and 1440 min after exposure. **(C)** For full spectrum p < 0.05 in comparison of Control (No GD) vs. GD/MDZ/CYS50 at 10–130 and 150–160 min after exposure; GD/MDZ/VEH vs. GD/MDZ/CYS50 at 140–1070 min after exposure. n = 11 for Control (No GD), n = 14 for GD/MDZ/VEH, n = 6 for GD/MDZ/CYS10, n = 9 for GD/MDZ/CYS50.

The percent of relative change from baseline levels in the power of (A) delta power band (0.1–4 Hz), (B) gamma power band (25.1–50 Hz), and (C) full spectrum power band are shown in Figure 3. All soman-exposed rats had an increase in percent change from baseline levels in delta power band (p < 0.05). Midazolam monotherapy delta levels returned to Control (No GD) levels briefly at 1240–1250, and 1270 min after exposure but otherwise had increased change from Control (No GD) levels for the full 24 h following exposure (p < 0.05). Rats treated with the 50 mg/kg dose of cystamine, when compared to midazolam monotherapy, had a greater initial increase in delta power band levels at 20, 50, and 110–170 min after exposure (p < 0.05) but had reduced change compared to midazolam monotherapy at 280–880 min after exposure (p < 0.05). All soman-exposed rats had an initial reduction in percent change in gamma power band compared to Control (No GD) levels (p < 0.05). Change in gamma power band levels for rats treated with the 50 mg/kg dose of cystamine was increased compared to midazolam monotherapy at 260–1070, 1140–1150, 1210, 1230, 1260–1270, 1300, 1320, 1340–1350, 1370, 1400, 1420, and 1440 min after exposure (p < 0.05). All soman-exposed rats had an initial increase in full spectrum power band compared to Control (No GD) levels (p < 0.05). Rats treated with the 50 mg/kg dose of cystamine returned to Control (No GD) levels earlier (at 170 min after exposure) than other treatment groups (p < 0.05); cystamine (10 mg/kg) treatment returned to Control (No GD) levels at 720 min after exposure (p < 0.05) and midazolam monotherapy treatment group returned to Control (No GD) levels at 1100 min after exposure (p < 0.05). Change in full spectrum power band levels was significantly reduced for rats treated with cystamine 50 mg/kg compared with midazolam monotherapy, 140–1070 min after soman exposure (p < 0.05).

**FIGURE 4:**
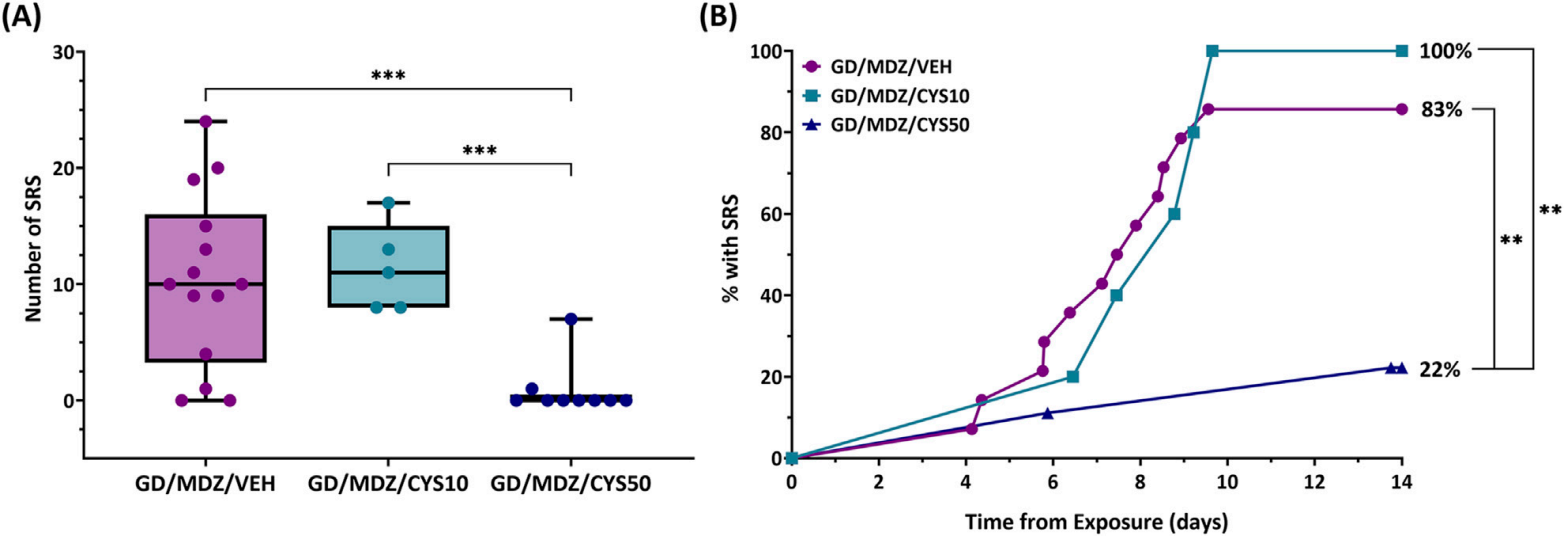
The effect of cystamine on the development of spontaneous recurrent seizures (SRS) following soman exposure in adult male rats. Rats exposed to soman were treated with atropine sulfate and HI-6 1 minute after exposure and with midazolam (GD/MDZ/VEH) with or without 10 mg/kg cystamine (GD/MDZ/CYS/10) or 50 mg/kg cystamine (GD/MDZ/CYS50) 30 min and 4 h after seizure onset. **(A)** Rats in the GD/MDZ/CYS50 group had fewer SRS occurrences compared to the GD/MDZ/VEH group and GD/MDZ/CYS10 group (***p < 0.001). n = 14 for GD/MDZ/VEH, n = 5 for GD/MDZ/CYS10, n = 9 for GD/MDZ/CYS50. **(B)** Onset of epileptogenesis is shown over a 14-day period from exposure. An effect of group was found on the median onset of SRS when comparing the GD/MDZ/CYS50 group to the GD/MDZ/VEH group (p < 0.01). The GD/MDZ/CYS50 group had a smaller percentage of rats that developed SRS compared to GD/MDZ/VEH and to GD/MDZ/CYS50. %SRS (**p < 0.01 at final % of animals with SRS). n = 14 for GD/MDZ/VEH, n = 5 for GD/MDZ/CYS10, n = 9 for GD/MDZ/CYS50.

Continuous monitoring of EEG data for the 14-day duration following soman exposure allowed for detection of spontaneous recurrent seizures (SRS). Rats that received the 50 mg/kg cystamine dose adjunct with midazolam had a lower number of SRS events compared to rats treated with the monotherapy and 10 mg/kg dual therapy 14 days after exposure (p < 0.001; Figure 4A). All treatment groups contained at least one subject that developed SRS approximately 4–6 days after exposure. While 100% of rats in the 10 mg/kg dual-therapy group developed SRS, the 50 mg/kg dual-therapy group had a substantial reduction in incidence, with only about 22% of rats affected (p < 0.01; Figure 4B).

### Neuronal loss and microglial activation after soman exposure

**FIGURE 5:**
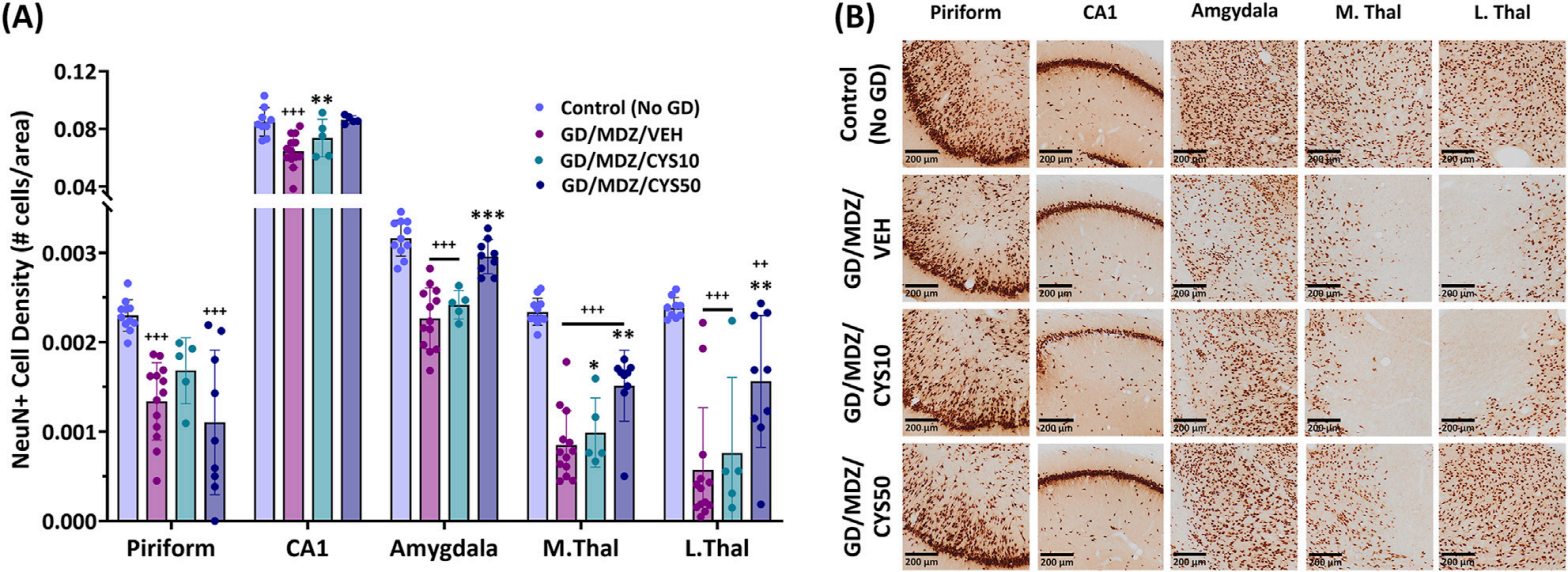
Soman exposure in adult male rats induced widespread neuronal loss in the piriform cortex, CA1 of the hippocampus, basolateral amygdala, medial thalamus (M. Thal), and lateral thalamus (L. Thal), shown here 2 weeks after exposure. Comparisons are made between Control (No GD), midazolam treatment (GD/MDZ/VEH), and combination midazolam and cystamine treatment groups (GD/MDZ/CYS10; GD/MDZ/CYS50). **(A)** Rats treated with the 50 mg/kg cystamine dose adjunct with midazolam had reduced neuronal loss in the CA1, amygdala, M. Thal, and L. Thal compared to midazolam monotherapy. Compared to GD/MDZ/VEH: **p < 0.01; ***p < 0.001. Compared to Control (No GD): ++p < 0.01; +++p < 0.001. n = 11 for Control (No GD), n = 14 for GD/MDZ/VEH, n = 5 for GD/MDZ/CYS10, n = 9 for GD/MDZ/CYS50. Data are shown as mean ± SD. **(B)** Representative photomicrographs of NeuN-stained coronal sections are shown at the 14-day endpoint depicting the piriform, CA1, amygdala, M. Thal, and L. Thal. Images are taken at ×10 magnification (scale bar = 200 µm).

Neuronal loss was demonstrated by a lower density of NeuN+ cells. Exposure to soman led to varying degrees of neuronal loss in the regions of interest across all groups (Figure 5A). Adjunct treatment with the 50 mg/kg cystamine dose significantly reduced neuronal loss in the CA1, amygdala, medial thalamus, and the lateral thalamus, compared to midazolam monotherapy (p < 0.01, p < 0.001). The 10 mg/kg dose did not afford significant neuroprotection overall. Representative photomicrographs of NeuN-stained coronal sections visualize treatment group differences in the piriform cortex, CA1 region of the hippocampus, amygdala, medial thalamus, and lateral thalamus (Figure 5B). Sparsely stained areas reflect the absence of neurons and greater damage, in contrast to denser or darker-stained regions where neuronal populations were better preserved; this difference was most pronounced when comparing the brain regions of the 50 mg/kg cystamine and monotherapy groups.

**FIGURE 6:**
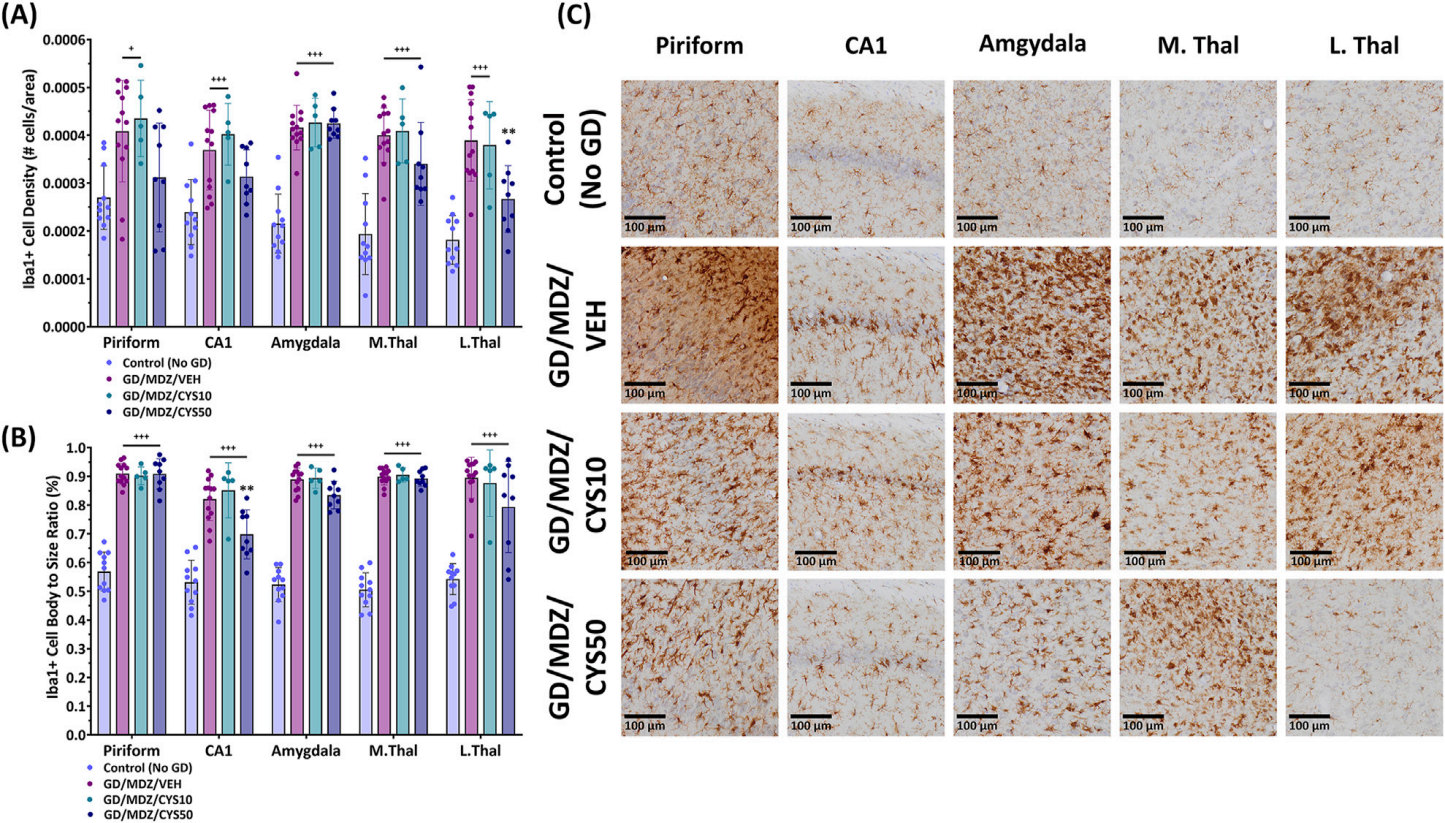
Soman exposure in adult male rats induced a severe neuroinflammatory response in the piriform cortex, CA1 of the hippocampus, basolateral amygdala, medial thalamus (M.Thal), and lateral thalamus (L.Thal), demonstrated with Iba+-staining. Comparisons are made between Control (No GD), midazolam (GD/MDZ/VEH), and combination midazolam and cystamine treatment groups (GD/MDZ/CYS10; GD/MDZ/CYS50). The GD/MDZ/CYS50 group **(A)** attenuated soman-induced increase in microglia density in the L. Thal and **(B)** mitigated microglial activation in the CA1 compared to the GD/MDZ/VEH group (**p < 0.01). The 10 mg/kg dose did not demonstrate significant protection against neuroinflammatory responses. Compared to GD/MDZ/VEH: **p < 0.01; Compared to Control (No GD): +p < 0.05; +++p < 0.001. n = 11 for Control (No GD), n = 14 for GD/MDZ, n = 5 for GD/MDZ/CYS10, n = 9 for GD/MDZ/CYS50. Data are shown as mean ± SD. **(C)** Representative photomicrographs of Iba1-stained coronal sections with a cresyl violet counterstain are shown at the 14-day endpoint depicting the piriform, CA1, amygdala, M. Thal, and L. Thal. Images were taken at ×20 magnification (scale bar = 100 µm).

An increase in Iba1+ cell density represents an increase in cell proliferation and an increase in cell body-to-size ratio represents the morphological changes that resident microglia undergo in response to seizure activity and central nervous system (CNS) damage. Varying degrees of microglial activation and proliferation were observed in animals treated with either the midazolam monotherapy or the midazolam-cystamine dual therapies based on qualitative observations of Iba1+ cell density and Iba1+ cell body-to-size ratio (Figures 6A,B). Adjunct treatment with the 50 mg/kg cystamine dose reduced soman-induced increase in Iba1+ cell density in the lateral thalamus compared to midazolam monotherapy (p < 0.01). This dose also decreased cell body-to-size ratio in the CA1 of the hippocampus compared to midazolam-treated rats after soman exposure (p < 0.01). The 10 mg/kg dose did not afford significant mitigation of microglial activation and proliferation. Representative photomicrographs of Iba1-stained coronal sections with a cresyl violet co-stain visualize treatment group differences in the piriform cortex, CA1 region of the hippocampus, amygdala, medial thalamus, and lateral thalamus (Figure 6C). An increased number of microglial cells and their aggregation, along with visibly enlarged cell bodies, indicate heightened activation in response to damage, whereas fewer cells with less density and reduced enlargement suggest attenuation of that response. This difference is most apparent when comparing the brain regions of the 50 mg/kg cystamine and monotherapy groups.

## Discussion

This study utilized a rodent model of soman-induced status epilepticus to investigate the therapeutic potential of an aminothiol in mitigating neurodegeneration. Compared to midazolam monotherapy, the midazolam and cystamine dual therapy demonstrated anti-seizure effects following soman exposure as observed in its reduction of initial time spent in seizure, fewer occurrences of SRS, reduced EEG power integral, and modulation of power band spectra, which may indicate reduced seizure severity. Furthermore, cystamine as adjunct to midazolam treatment ameliorated neuronal damage and suppressed microglial activation, which occurs following exposure to a seizure-inducing dose of soman in rodents [22, 36, 44, 45].

The higher dose of cystamine (50 mg/kg) in combination with midazolam was more effective than the lower dose (10 mg/kg) in reducing soman-induced status epilepticus, specifically by reducing the total time spent in initial seizures as well as a lower occurrence and percentage of rats that developed SRS, compared to midazolam monotherapy. The duration of initial seizure was associated with the extent of neurodegeneration, with less time in seizure resulting in less brain damage [16]. The development of SRS after soman exposure in rats treated with a benzodiazepine is associated with greater hippocampal damage compared to rats that do not develop SRS [46]. Greater suppression of soman-induced seizure duration and reduction in SRS development by cystamine (50 mg/kg) as an adjunct to midazolam likely contributed to reduced neuronal loss compared to rats treated with midazolam only. The longer time in seizure and high occurrences of SRS may have contributed to the greater neuropathology observed in animals that received the lower dose (10 mg/kg) of cystamine. In addition, attenuation of soman-induced increases in delta band activity, as observed in our findings with rats treated with the cystamine 50 mg/kg dose, corresponded to less neurodegeneration [47, 48]. It is important to note however, extensive neurodegeneration can still occur in the absence of recurrent seizures, possibly driven by mechanisms such as excitotoxicity from initial seizure events or inflammation that persists beyond the initial trauma after exposure [17].

Cystamine as adjunct therapy to midazolam reduced neurodegeneration in the CA1, amygdala, and regions of the thalamus. Immunohistochemical analysis of our findings showing reduced neuronal loss and reduced microglia proliferation activity in the CA1 of rats that received the 50 mg/kg dual treatment presents an intriguing insight about cystamine’s mechanism of action. The hippocampus, which encompasses the CA1 subregion, is one of the seizure-sensitive brain regions most vulnerable to severe neuronal damage after nerve agent exposure [24, 49, 50]. An excessive reactive microglial response, as observed in other models of nerve agent-induced toxicity [44], can exacerbate neurodegeneration, where these immune cells can contribute to neuronal injury through the release of pro-inflammatory cytokines and reactive oxygen species (ROS) [51, 52]. This aligns with broader observations in neurodegenerative disease such as Alzheimer’s and Parkinson’s disease, where chronic neuroinflammation and dysregulated glial activity are hallmarks of disease progression [53–55]. By reducing the degree of microglial activation in soman-exposed rats, it is possible that cystamine as an adjunct to midazolam is also reducing the exacerbation of neuronal degeneration. Although the current study shows promising results, its short-term duration (2 weeks) offers only a preliminary understanding. Further studies are needed to investigate the long-term neuroprotective effects of treatment with aminothiols cystamine and cysteamine in models of status epilepticus, as well as to assess the efficacy of sub-chronic and chronic treatment with aminothiols in these models. In addition, future studies would benefit from a larger sample size, especially in cases of reduced survivors to soman exposure.

The precise mechanism of action of cystamine is not fully understood, but evidence suggests its protective effects are largely indirect. Cystamine is rapidly metabolized into cysteamine, resulting in the accumulation of the cellular antioxidant cysteine [1, 56, 57]. The accumulation of cysteine (also proposed to be neuroprotective) supports the synthesis of glutathione, another powerful antioxidant that helps counteract oxidative stress via the neutralization of ROS [56, 58, 59]. Beyond its influence on antioxidant levels, cystamine metabolites are thought to interact with several neuroprotective pathways. Notably, cystamine has been shown to inhibit transglutaminase, an enzyme implicated in the neurodegenerative process and cell death, where it catalyzes the crosslinking of toxic protein aggregates during times of stress [60]. Additionally, cysteamine, the active form of cystamine, has been linked to an increase in BDNF levels, which has been shown to play a crucial role in neuronal survival and plasticity [2, 5, 6]. Interestingly, our laboratory previously observed that low dose exposure to the nerve agent O-ethyl-S-(2-diisopropylaminoethyl)-methylphosphonothiolate (VX) in mice increased BDNF in CA3 of the hippocampus and suggested that elevated BDNF may have been an adaptive response in this model [61]. Overall, it appears that cystamine potentially triggered a cascade of biochemical changes that collectively bolstered neuronal resilience against damage.

Our findings reinforce existing evidence of cystamine’s neuroprotective potential. Cystamine has demonstrated efficacy in other models of neurotoxicity, including 3-nitropropionic (3-NP), 6-hydroxydopamine (6-OHDA), and 1-methyl-4-phenyl-1,2,3,6-tetrahydropyridine (MPTP), which resulted in damage to the substantia nigra and striatum [6, 7, 9]. Like the brain regions examined in this study, substantia nigra and striatum are highly susceptible to severe neuronal damage following exposure [62–64]. For instance, in a 3-NP model, cystamine reduced striatal lesion size [7], while in 6-OHDA and MPTP preclinical models of Parkinson’s disease it mitigated neuronal loss in the substantia nigra and preserved dopaminergic projections to the striatum [9]. Moreover, cysteamine, an analog of cystamine, exhibited a dose-dependent protection of striatal dopaminergic neurons and substantia nigra neurons in a mouse model of neurodegeneration [6]. Comparable effects of cystamine treatment on neuroprotection were observed in our findings where viable neurons were preserved, and reactive microglia response was attenuated in seizure sensitive brain regions following soman exposure. Although the toxicity-induced models cited here differ in their mechanisms of toxicity from soman, there may be overlap in the secondary mechanisms of cell death such as oxidative stress, which may be protected by treatment with cystamine. Additionally, while the brain regions of interest we report here differ from those investigated in the aforementioned studies, they share susceptibility to neurotoxic damage, further supporting cystamine’s therapeutic potential across diverse neural systems and regions.

Neuronal injury often results in the integrity of the blood brain barrier (BBB) being compromised, as reported in epilepsy models from a multitude of etiologies [65] and in nerve agent animal models [66]. This phenomenon allows for the recruitment of peripheral immune cells in the brain with less resistance [67]. Despite this impairment of BBB integrity, the BBB itself is still a significant challenge to the clinical application of many neuroactive compounds. Cystamine, through its reduced form, cysteamine, has been reported to effectively cross the blood brain barrier, where it has been shown to enhance neuronal survival through its mitigation of mitochondrial dysfunction [1], which is central to the pathophysiology of many neurodegenerative conditions. Bousquet et al., (2010) [3] reports cystamine facilitates cysteamine brain transport as evidenced by their findings in determining the brain transport coefficient which indicates the degree of brain uptake. It is possible that infiltration of peripheral immune cells in the brain to assist with the neuroinflammatory response, coupled with cystamine’s neuroprotective mechanisms is why less neurodegeneration is observed in animals treated with the cystamine dual therapies. Further studies are needed to better understand cystamine’s long-term effects on the BBB in the context of nerve agent-induced status epilepticus as well as other neurodegenerative disease models.

Much of the existing research regarding organophosphorus nerve agents primarily focused on management of seizures and therefore limits attention to the underlying neurodegeneration, neuroinflammation, and excitotoxicity that can persist after exposure. Although our focus was on neuroprotective potential of cystamine, as an adjunct treatment with midazolam our present finding of reduced time in seizure and reduced incidence of SRS may have contributed to our observed neuroprotective findings in rats treated with cystamine and midazolam, over midazolam monotherapy. Whether the multi-faceted neuroprotective mechanisms of action of cystamine [1] provided added benefit over that of reduction in initial seizure requires further study. The neuroprotective efficacy of cystamine as adjunct to midazolam tested in this study against soman-induced toxicity offers a promising approach to this challenge and extends the current understanding of the neuroprotective potential of aminothiols. Managing oxidative stress caused by soman-induced toxicity with cystamine may reduce the overactivation of microglia, thereby limiting their potentially damaging effects and allowing for a more reparative neuroimmune response. Furthermore, since cystamine influences metabolic pathways, further research should explore whether treatments to endogenously enhance these natural processes might provide a sustained and multifaceted defense against cell death.

## Data Availability

The raw data supporting the conclusions of this article will be made available by the authors, without undue reservation.

## References

[1] Paul BD Snyder SH. Therapeutic applications of cysteamine and cystamine in neurodegenerative and neuropsychiatric diseases. Front Neurol (2019) 10:1315. 10.3389/fneur.2019.01315 31920936 PMC6920251

[2] Borrell-Pages M Canals JM Cordelieres FP Parker JA Pineda JR Grange G Cystamine and cysteamine increase brain levels of BDNF in Huntington disease via HSJ1b and transglutaminase. J Clin Invest (2006) 116:1410–24. 10.1172/JCI27607 16604191 PMC1430359

[3] Bousquet M Gibrat C Ouellet M Rouillard C Calon F Cicchetti F. Cystamine metabolism and brain transport properties: clinical implications for neurodegenerative diseases. J Neurochem (2010) 114:1651–8. 10.1111/j.1471-4159.2010.06874.x 20569301

[4] Arbez N Roby E Akimov S Eddings C Ren M Wang X Cysteamine protects neurons from mutant huntingtin toxicity. J Huntington's Dis (2019) 8:129–43. 10.3233/jhd-180312 30856117 PMC7039181

[5] Gibrat C Bousquet M Saint-Pierre M Levesque D Calon F Rouillard C Cystamine prevents MPTP-induced toxicity in young adult mice via the up-regulation of the brain-derived neurotrophic factor. Prog Neuro-Psychopharmacology Biol Psychiatry (2010) 34:193–203. 10.1016/j.pnpbp.2009.11.005 19913065

[6] Sun L Xu S Zhou M Wang C Wu Y Chan P. Effects of cysteamine on MPTP-induced dopaminergic neurodegeneration in mice. Brain Res (2010) 1335:74–82. 10.1016/j.brainres.2010.03.079 20380823

[7] Calkins MJ Townsend JA Johnson DA Johnson JA. Cystamine protects from 3-nitropropionic acid lesioning via induction of nf-e2 related factor 2 mediated transcription. Exp Neurol (2010) 224:307–17. 10.1016/j.expneurol.2010.04.008 20406637 PMC2885467

[8] Cisbani G Drouin-Ouellet J Gibrat C Saint-Pierre M Lagace M Badrinarayanan S Cystamine/cysteamine rescues the dopaminergic system and shows neurorestorative properties in an animal model of Parkinson's disease. Neurobiol Dis (2015) 82:430–44. 10.1016/j.nbd.2015.07.012 26232588

[9] Stack EC Ferro JL Kim J Del Signore SJ Goodrich S Matson S Therapeutic attenuation of mitochondrial dysfunction and oxidative stress in neurotoxin models of Parkinson's disease. Biochim Biophys Acta (Bba) - Mol Basis Dis (2008) 1782:151–62. 10.1016/j.bbadis.2007.12.006 18206128

[10] Cicchetti F David LS Siddu A Denis HL. Cysteamine as a novel disease-modifying compound for Parkinson's disease: over a decade of research supporting a clinical trial. Neurobiol Dis (2019) 130:104530. 10.1016/j.nbd.2019.104530 31301344

[11] Hulse EJ Haslam JD Emmett SR Woolley T. Organophosphorus nerve agent poisoning: managing the poisoned patient. Br J Anaesth (2019) 123:457–63. 10.1016/j.bja.2019.04.061 31248646

[12] Savolainen K. CHAPTER 50 - understanding the toxic actions of organophosphates. (2001).

[13] Reddy DS. Chapter 20 - advances in targeted therapy of organophosphate neurotoxicity and chemical warfare nerve agents. (2023).

[14] Kadar T Cohen G Sahar R Alkalai D Shapira S. Long-term study of brain lesions following soman, in comparison to DFP and metrazol poisoning. Hum Exp Toxicol (1992) 11:517–23. 10.1177/096032719201100613 1361142

[15] Petras JM. Neurology and neuropathology of Soman-induced brain injury: an overview. J Exp Anal Behav (1994) 61:319–29. 10.1901/jeab.1994.61-319 8169578 PMC1334419

[16] McDonough JH Jr Shih TM. Neuropharmacological mechanisms of nerve agent-induced seizure and neuropathology 1 1The animals used in studies performed in, or sponsored by, this Institute were handled in accordance with the principles stated in the Guide for the Care and use of laboratory animals, proposed by the committee on Care and use of laboratory animals of the Institute of laboratory animal resources, national research council, DHHA, national Institute of health publication #85–23, 1985, and the animal Welfare Act of 1966, as amended. The opinions, or assertions contained herein are the private views of the authors, and are not to be construed as reflecting the views of the department of the Army or the department of defense. Neurosci & Biobehavioral Rev (1997) 21:559–79. 10.1016/s0149-7634(96)00050-4 9353792

[17] Chen Y. Organophosphate-induced brain damage: mechanisms, neuropsychiatric and neurological consequences, and potential therapeutic strategies. Neurotoxicology (2012) 33:391–400. 10.1016/j.neuro.2012.03.011 22498093

[18] de Araujo Furtado M Lumley LA Robison C Tong LC Lichtenstein S Yourick DL. Spontaneous recurrent seizures after status epilepticus induced by soman in Sprague-Dawley rats. Epilepsia (2010) 51:1503–10. 10.1111/j.1528-1167.2009.02478.x 20067510

[19] Jett DA Sibrizzi CA Blain RB Hartman PA Lein PJ Taylor KW A national toxicology program systematic review of the evidence for long-term effects after acute exposure to sarin nerve agent. Crit Rev Toxicol (2020) 50:474–90. 10.1080/10408444.2020.1787330 32755358 PMC8011809

[20] Cannard K. The acute treatment of nerve agent exposure. J Neurol Sci (2006) 249:86–94. 10.1016/j.jns.2006.06.008 16945386

[21] Newmark J. Therapy for acute nerve agent poisoning: an update. Neurol Clin Pract (2019) 9:337–42. 10.1212/cpj.0000000000000641 31583189 PMC6745742

[22] Marrero‐Rosado B de Araujo Furtado M Schultz CR Stone M Kundrick E Walker K Soman-induced status epilepticus, epileptogenesis, and neuropathology in carboxylesterase knockout mice treated with midazolam. Epilepsia (2018) 59:2206–18. 10.1111/epi.14582 30368799 PMC6334636

[23] Reddy SD Reddy DS. Midazolam as an anticonvulsant antidote for organophosphate intoxication--A pharmacotherapeutic appraisal. Epilepsia (2015) 56:813–21. 10.1111/epi.12989 26032507 PMC4457669

[24] Shih TM Duniho SM McDonough JH. Control of nerve agent-induced seizures is critical for neuroprotection and survival. Toxicol Appl Pharmacol (2003) 188:69–80. 10.1016/s0041-008x(03)00019-x 12691725

[25] Shih TM McDonough JH. Efficacy of biperiden and atropine as anticonvulsant treatment for organophosphorus nerve agent intoxication. Arch Toxicol (2000) 74:165–72. 10.1007/s002040050670 10877003

[26] Petras JM. Soman neurotoxicity. Toxicol Sci (1981) 1:242. 10.1093/toxsci/1.2.242 7184789

[27] Shih TM McDonough JH Jr. Neurochemical mechanisms in soman-induced seizures. J Appl Toxicol (1997) 17:255–64. 10.1002/(sici)1099-1263(199707)17:4<255::aid-jat441>3.3.co;2-4 9285539

[28] Shih TM McDonough JH Jr. Organophosphorus nerve agents-induced seizures and efficacy of atropine sulfate as anticonvulsant treatment. Pharmacol Biochem Behav (1999) 64:147–53. 10.1016/s0091-3057(99)00114-8 10495009

[29] Talbot BG Anderson DR Harris LW Yarbrough LW Lermox WJ. A comparison of *in vivo* and *in vitro* rates of aging of soman-inhibited erythrocyte acetylcholinesterase in different animal species. Drug Chem Toxicol (1988) 11:289–305. 10.3109/01480548809017884 3181042

[30] Tsung-Ming S Whalley CE Valdes JJ. A comparison of cholinergic effects of HI-6 and pralidoxime-2-chloride (2-PAM) in soman poisoning. Toxicol Lett (1991) 55:131–47. 10.1016/0378-4274(91)90128-s 1998202

[31] McDonough JH McMonagle JD Shih TM. Time-dependent reduction in the anticonvulsant effectiveness of diazepam against soman-induced seizures in Guinea pigs. Drug Chem Toxicol (2010) 33:279–83. 10.3109/01480540903483417 20429808

[32] Shih T McDonough JH Jr Koplovitz I. Anticonvulsants for soman-induced seizure activity. J Biomed Sci (1999) 6:86–96. 10.1007/bf02256439 10087439

[33] Moffett MC Schultz MK Schwartz JE Stone MF Lumley LA. Impaired auditory and contextual fear conditioning in soman-exposed rats. Pharmacol Biochem Behav (2011) 98:120–9. 10.1016/j.pbb.2010.11.022 21144858

[34] Rossetti F de Araujo Furtado M Pak T Bailey K Shields M Chanda S Combined diazepam and HDAC inhibitor treatment protects against seizures and neuronal damage caused by soman exposure. Neurotoxicology (2012) 33:500–11. 10.1016/j.neuro.2012.02.010 22387230

[35] Lumley LA Marrero‐Rosado B Rossetti F Schultz CR Stone MF Niquet J Combination of antiseizure medications phenobarbital, ketamine, and midazolam reduces soman-induced epileptogenesis and brain pathology in rats. Epilepsia Open (2021) 6:757–69. 10.1002/epi4.12552 34657398 PMC8633481

[36] Nguyen DA Stone MF Schultz CR de Araujo Furtado M Niquet J Wasterlain CG Evaluation of midazolam-ketamine-allopregnanolone combination therapy against cholinergic-induced status epilepticus in rats. The J Pharmacol Exp Ther (2024) 388:376–85. 10.1124/jpet.123.001784 37770198 PMC10801769

[37] Wright LK Lee RB Vincelli NM Whalley CE Lumley LA. Comparison of the lethal effects of chemical warfare nerve agents across multiple ages. Toxicol Lett (2016) 241:167–74. 10.1016/j.toxlet.2015.11.023 26621540

[38] Tremblay ME Saint-Pierre M Bourhis E Lévesque D Rouillard C Cicchetti F. Neuroprotective effects of cystamine in aged parkinsonian mice. Neurobiol Aging (2006) 27:862–70. 10.1016/j.neurobiolaging.2005.04.004 15913845

[39] Wang S Li X Li M Jiang L Yuan H Han W Cystamine attenuated behavioral deficiency via increasing the expression of BDNF and activating PI3K/Akt signaling in 2,5-hexanedione intoxicated rats. Toxicol Res (Camb) (2017) 6:199–204. 10.1039/c6tx00409a 30090490 PMC6062339

[40] de Araujo Furtado M Zheng A Sedigh-Sarvestani M Lumley L Lichtenstein S Yourick D. Analyzing large data sets acquired through telemetry from rats exposed to organophosphorous compounds: an EEG study. J Neurosci Methods (2009) 184:176–83. 10.1016/j.jneumeth.2009.07.020 19632275

[41] Lumley LA Rossetti F de Araujo Furtado M Marrero-Rosado B Schultz CR Schultz MK Dataset of EEG power integral, spontaneous recurrent seizure and behavioral responses following combination drug therapy in soman-exposed rats. Data in Brief (2019) 27:104629. 10.1016/j.dib.2019.104629 31687442 PMC6820070

[42] Niquet J Baldwin R Norman K Suchomelova L Lumley L Wasterlain CG. Midazolam-ketamine dual therapy stops cholinergic status epilepticus and reduces Morris water maze deficits. Epilepsia (2016) 57:1406–15. 10.1111/epi.13480 27500978 PMC5012923

[43] Racine RJ Burnham WM Gartner JG. First trial motor seizures triggered by amygdaloid stimulation in the rat. Electroencephalography Clin Neurophysiol (1973) 35:487–94. 10.1016/0013-4694(73)90024-2 4126454

[44] Lumley LA Nguyen DA de Araujo Furtado M Niquet J Linz EO Schultz CR Efficacy of lacosamide and rufinamide as adjuncts to midazolam-ketamine treatment against cholinergic-induced status epilepticus in rats. The J Pharmacol Exp Ther (2024) 388:347–57. 10.1124/jpet.123.001789 37977809 PMC10801783

[45] Reddy DS. Mechanism-based novel antidotes for organophosphate neurotoxicity. Curr Opin Toxicol (2019) 14:35–45. 10.1016/j.cotox.2019.08.001 32856007 PMC7448382

[46] de Araujo Furtado M Rossetti F Chanda S Yourick D. Exposure to nerve agents: from status epilepticus to neuroinflammation, brain damage, neurogenesis and epilepsy. Neurotoxicology (2012) 33:1476–90. 10.1016/j.neuro.2012.09.001 23000013

[47] Carpentier P Foquin A Kamenka JM Rondouin G Lerner-Natoli M de Groot DM Effects of thienylphencyclidine (TCP) on seizure activity and brain damage produced by soman in Guinea-pigs: ECoG correlates of neurotoxicity. Neurotoxicology (2001) 22:13–28. 10.1016/s0161-813x(00)00016-4 11307847

[48] McDonough JH Jr Clark TR Slone TW Jr Zoeffel D Brown K Kim S Neural lesions in the rat and their relationship to EEG delta activity following seizures induced by the nerve agent soman. Neurotoxicology (1998) 19:381–91. 9621344

[49] Apland JP Figueiredo TH Qashu F Aroniadou-Anderjaska V Souza AP Braga MF. Higher susceptibility of the ventral versus the dorsal hippocampus and the posteroventral versus anterodorsal amygdala to soman-induced neuropathology. Neurotoxicology (2010) 31:485–92. 10.1016/j.neuro.2010.05.014 20570628 PMC2933957

[50] McDonough JH Jr Jaax NK Crowley RA Mays MZ Modrow HE. Atropine and/or diazepam therapy protects against soman-induced neural and cardiac pathology. Toxicol Sci (1989) 13:256–76. 10.1093/toxsci/13.2.256 2792594

[51] Simpson DSA Oliver PL. ROS generation in microglia: understanding oxidative stress and inflammation in neurodegenerative disease. Antioxidants (Basel) (2020) 9:743. 10.3390/antiox9080743 32823544 PMC7463655

[52] Streit WJ Mrak RE Griffin WS. Microglia and neuroinflammation: a pathological perspective. J Neuroinflammation (2004) 1:14. 10.1186/1742-2094-1-14 15285801 PMC509427

[53] Gao C Jiang J Tan Y Chen S. Microglia in neurodegenerative diseases: mechanism and potential therapeutic targets. Signal Transduction Targeted Ther (2023) 8:359. 10.1038/s41392-023-01588-0 PMC10514343 37735487

[54] Isik S Yeman Kiyak B Akbayir R Seyhali R Arpaci T. Microglia mediated neuroinflammation in Parkinson's disease. Cells (2023) 12:1012. 10.3390/cells12071012 37048085 PMC10093562

[55] Cai Y Liu J Wang B Sun M Yang H. Microglia in the neuroinflammatory pathogenesis of alzheimer's disease and related therapeutic targets. Front Immunol (2022) 13:856376. 10.3389/fimmu.2022.856376 35558075 PMC9086828

[56] Fox JH Barber DS Singh B Zucker B Swindell MK Norflus F Cystamine increases L-cysteine levels in Huntington's disease transgenic mouse brain and in a PC12 model of polyglutamine aggregation. J Neurochem (2004) 91:413–22. 10.1111/j.1471-4159.2004.02726.x 15447674

[57] Pinto JT Van Raamsdonk JM Leavitt BR Hayden MR Jeitner TM Thaler HT Treatment of YAC128 mice and their wild-type littermates with cystamine does not lead to its accumulation in plasma or brain: implications for the treatment of Huntington disease. J Neurochem (2005) 94:1087–101. 10.1111/j.1471-4159.2005.03255.x 15992377

[58] Jokay I Kelemenics K Gyuris A Minarovits J. S-methylthio-cysteine and cystamine are potent stimulators of thiol production and glutathione synthesis. Life Sci (1997) 62:PL27–33. 10.1016/s0024-3205(97)01066-7 9488118

[59] Lesort M Lee M Tucholski J Johnson GV. Cystamine inhibits caspase activity. J Biol Chem (2003) 278:3825–30. 10.1074/jbc.m205812200 12458211

[60] Jeitner TM Pinto JT Cooper AJL. Cystamine and cysteamine as inhibitors of transglutaminase activity *in vivo* . Biosci Rep (2018) 38. 10.1042/bsr20180691 PMC6123069 30054429

[61] Pizarro JM Chang WE Bah MJ Wright LK Saviolakis GA Alagappan A Repeated exposure to sublethal doses of the organophosphorus compound VX activates BDNF expression in mouse brain. Toxicol Sci (2012) 126:497–505. 10.1093/toxsci/kfr353 22240983

[62] Beal MF Brouillet E Jenkins BG Ferrante RJ Kowall NW Miller JM Neurochemical and histologic characterization of striatal excitotoxic lesions produced by the mitochondrial toxin 3-nitropropionic acid. J Neurosci (1993) 13:4181–92. 10.1523/jneurosci.13-10-04181.1993 7692009 PMC6576392

[63] Bossi SR Simpson JR Isacson O. Age dependence of striatal neuronal death caused by mitochondrial dysfunction. Neuroreport (1993) 4:73–6. 10.1097/00001756-199301000-00019 8453041

[64] Pandey M Borah A Varghese M Barman PK Mohanakumar KP Usha R. Striatal dopamine level contributes to hydroxyl radical generation and subsequent neurodegeneration in the striatum in 3-nitropropionic acid-induced Huntington's disease in rats. Neurochem Int (2009) 55:431–7. 10.1016/j.neuint.2009.04.013 19410615

[65] DePaula-Silva AB. The contribution of microglia and brain-infiltrating macrophages to the pathogenesis of neuroinflammatory and neurodegenerative diseases during TMEV infection of the central nervous system. Viruses (2024) 16:119. 10.3390/v16010119 38257819 PMC10819099

[66] Nguyen DA Niquet J Marrero-Rosado B Schultz CR Stone MF de Araujo Furtado M Age differences in organophosphorus nerve agent-induced seizure, blood brain barrier integrity, and neurodegeneration in midazolam-treated rats. Exp Neurol (2025) 385:115122. 10.1016/j.expneurol.2024.115122 39710244

[67] Chen T Dai Y Hu C Lin Z Wang S Yang J Cellular and molecular mechanisms of the blood-brain barrier dysfunction in neurodegenerative diseases. Fluids Barriers CNS (2024) 21:60. 10.1186/s12987-024-00557-1 39030617 PMC11264766

