# Supplementary figures and images for: Cystamine reduces neurodegeneration and epileptogenesis following soman-induced status epilepticus in rats
Source: Exp Biol Med (Maywood). 2025 Jun 9;250:10598. doi: 10.3389/ebm.2025.10598 (PMC12183515; doi:10.3389/ebm.2025.10598)

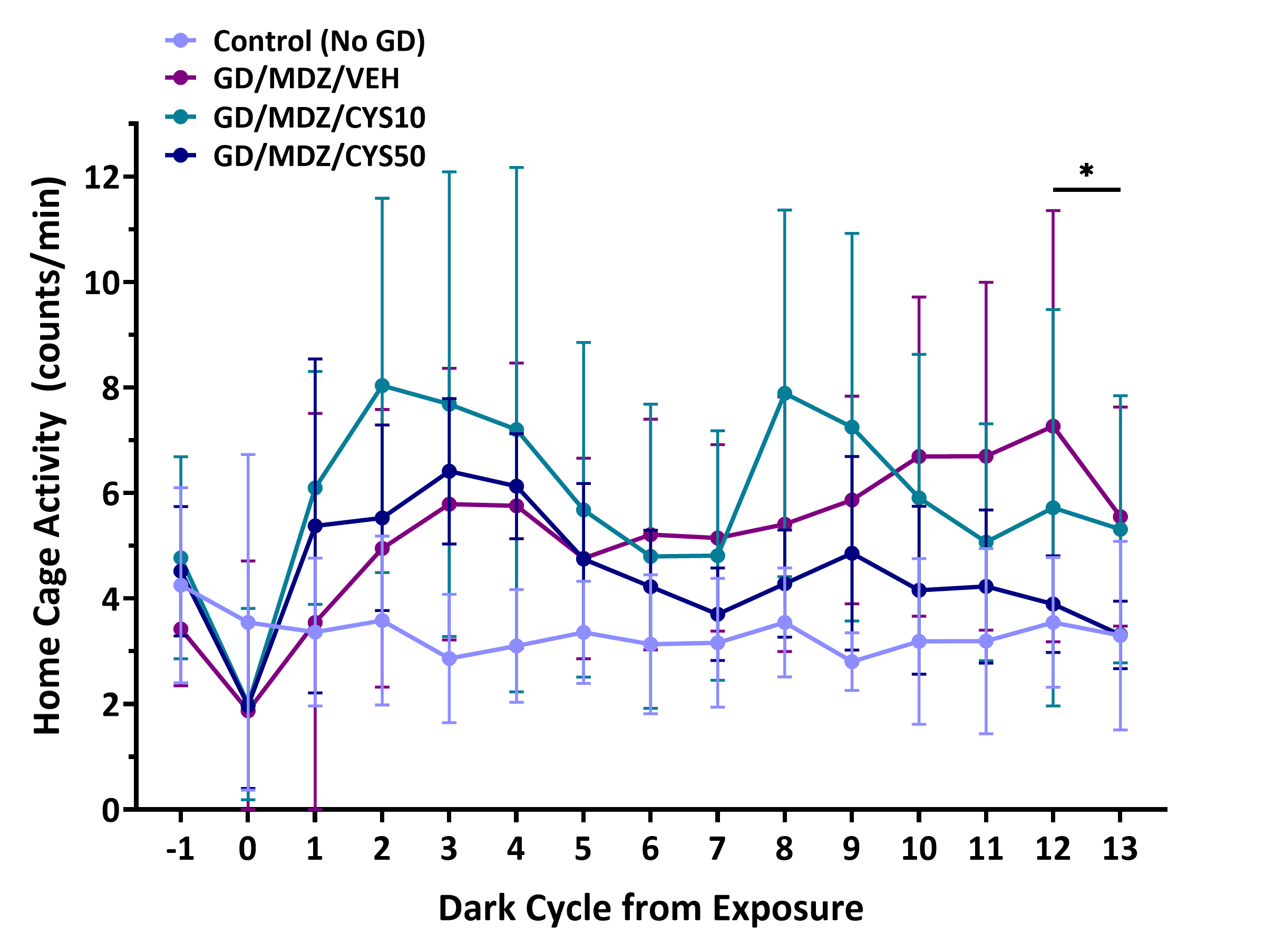

Supplement: Supplementary file 1 [file Image2.tif]

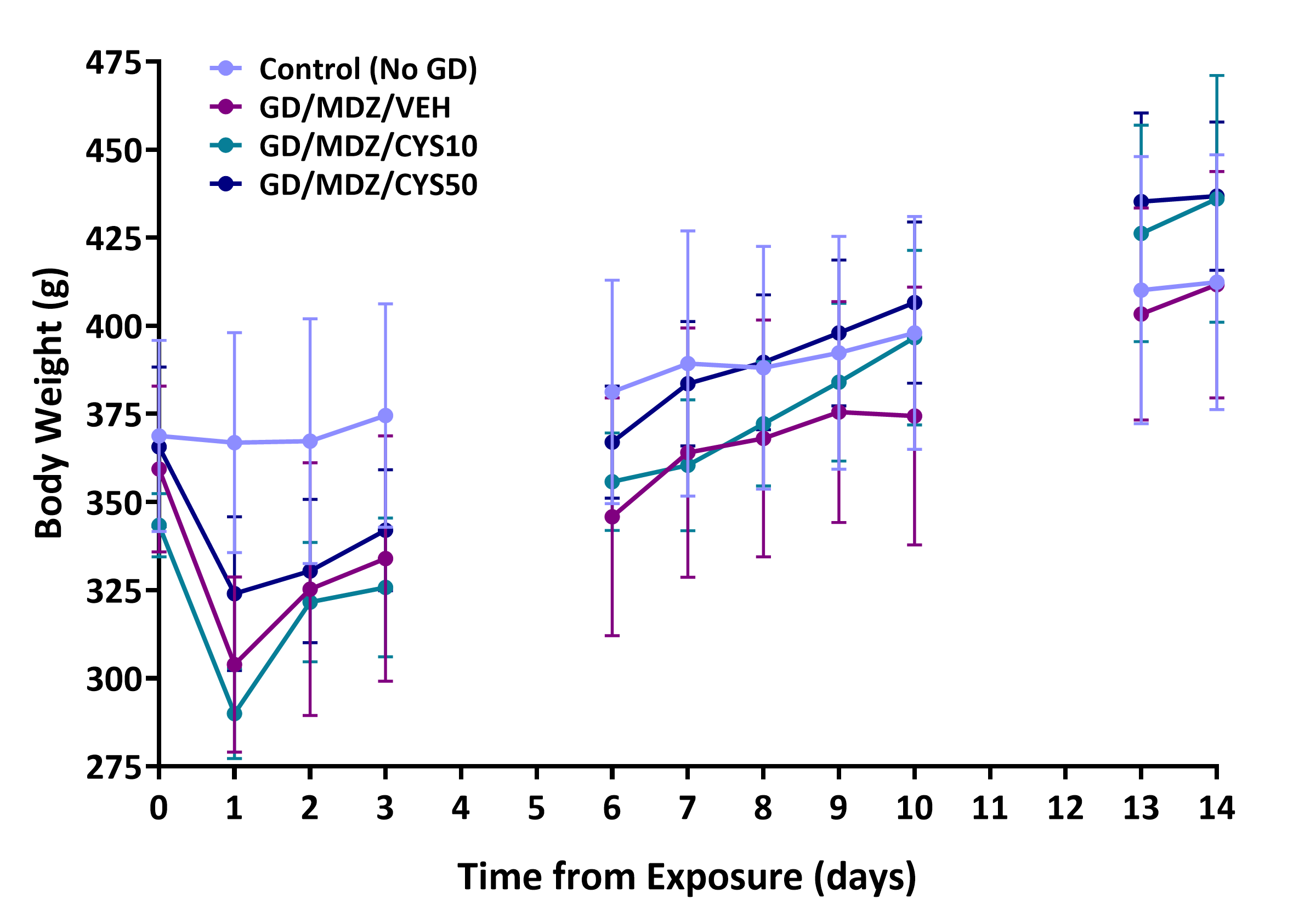

Supplement: Supplementary file 2 [file Image1.tif]
